# Outcome of Primary Cemented Bipolar Hemiarthroplasty compared with Dynamic Hip Screw in Elderly Patients with Unstable Intertrochanteric Fracture

**DOI:** 10.5704/MOJ.1803.007

**Published:** 2018-03

**Authors:** YN Gashi, AS Elhadi, IM Elbushra

**Affiliations:** Department of Orthopaedics, University of Khartoum, Khartoum, Sudan; ^*^Department of Orthopaedics, Ibrahim Malik Teaching Hospital, Khartoum, Sudan; ^**^Department of Orthopaedics, Best Care Hospital, Khartoum, Sudan

**Keywords:** unstable, intertrochanteric fracture, bipolar hemiarthroplasty, DHS

## Abstract

**Introduction:** Although the treatment of choice for unstable intertrochanteric fractures in elderly patients has been internal fixation for a long time, several studies have shown mechanical and technical failures. Primary cemented bipolar (PCB) hemiarthroplasty has been proposed as an alternative with some advantages concerning earlier mobilization and minimal postoperative complications.

**Materials and Methods:** This is a prospective cohort hospital-based study conducted at three tertiary hospitals over a period of two years. A total of 98 patients were enrolled in the study, 38 patients treated with Dynamic Hip Screw (DHS) and 60 patients treated with PCB hemiarthroplasty. Intraoperative events (e.g. duration of surgery and blood loss), hospital stay, weight bearing, Harris Hip score and post-operative complications were used as predictors of final outcome. Mean follow-up was 13.66±5.9 months in hemiarthroplasty group and 11.8±2.7 months at internal fixation group.

**Results:** The two groups were comparable in age, sex, comorbidity, mode of trauma, and classification of fracture. Early mobilisation was significantly better in hemiarthroplasty (p<0.001) where 93.3% of patients started partial weight bearing on postoperative Day 1, while in the DHS group, 73.7% of patients started partial weight bearing after two weeks postoperatively. At the final follow-up, the mortality rate did not differ between the two groups, but general and mechanical complications were more common in the DHS group. The mean Harris Hip score was better in the hemiarthroplasty group (91.14 vs 74.11).

**Conclusion:** Primary cemented bipolar hemiarthroplasty is a safe and valid option in treating unstable intertrochanteric fracture. Although it has been shown to have some advantages over DHS in certain circumstances, lack of randomization and difficulties in standardization of patients and treating surgeon raise a need for more studies with bigger sample size and proper randomization.

## Introduction

The management of unstable intertrochanteric fractures in elderly patients is a challenge because of the difficulty in obtaining anatomical reduction and association with high rates of morbidity and mortality. For several decades, the treatment of choice for unstable intertrochanteric fractures in elderly patients has been internal fixation, although, several studies have shown mechanical and technical failures^1-3^. Those failures were thought to be due to the use of extra-medullary implants, but a recent study showed no difference between intra- or extra-medullary types of fixation in unstable intertrochanteric fracture^4^. In our setting, DHS was used and still being used widely for treatment of both stable and unstable intertrochanteric hip fractures, but with a lot of complications especially in unstable fractures. Treatment with primary bipolar arthroplasty could perhaps return these patients to their preinjury level of activity more quickly, thus obviating the postoperative complications caused by immobilization or failure of the implant^5^.

The aim of this study was to compare the outcome of primary cemented bipolar (PCB) hemiarthroplasty with DHS in the management of comminuted intertrochanteric hip fractures in elderly patients.

## Materials and Methods

This is a prospective cohort hospital-based study conducted at three main tertiary hospitals, conducted over a period of two years (2014 to 2016). A total of 98 patients were enrolled in the study, all were above 65 years old with unstable fractures, and those with stable and pathological intertrochanteric fracture were excluded from this study. They were treated by orthopaedic surgeons with a minimum of three years’ experience in hip trauma. This is a total coverage of all patients presented during the study duration.

Thirty-eight patients were treated with DHS and 60 patients were treated with PCB hemiarthroplasty, the patients being allocated to either group according to hospital policy. Personal data, mode of trauma and comorbidity were recorded using a structured questionnaire. Fractures were classified according to Kyle classification^6^. Details about intraoperative events (e.g. duration of surgery and blood loss) were recorded. Partial weight bearing, hospital stay, full weight bearing, infection, and other complications were used as predictors of postoperative improvement and complications.

All patients received preoperative prophylactic antibiotics (1.5 mg of cefuroxime with induction of anaesthesia) and postoperative anticoagulant treatment (4000 IU of low-molecular-weight heparin). All patients were seen at two weeks, six weeks, and 12 weeks postoperatively, and at the final follow-up, which was 13.66±5.9 months in hemiarthroplasty group and 11.8±2.7 months at internal fixation group. All patients were evaluated using Harris Hip score at three months and at the final follow-up to assess functional outcomes.

Dynamic Hip Screw (DHS): On the traction table, through a direct lateral femoral approach with vastus lateralis reflection (majority of cases), the lag screw applied after reduction and its position checked with a C-arm. Tip apex distance (TAD) was taken into consideration. Thereafter, a 4-hole side plate [S.H Pitkar Pvt. Ltd, Pimpri Chinchwad, India] was fixed to the femoral shaft with cortical screws.

PCB hemiarthroplasty: All arthroplasties were performed through the lateral Hardinge approach in the decubitus position. The head and bony fragments were removed except for the greater trochanter. The calcar was removed and remodelled with cement in some cases. Thereafter, the greater trochanter was reattached with cerclage wire and a three-piece bipolar prosthesis was applied with cementation. The implant used was the LINK SP II hip prosthesis [Waldemar Link-Hamburg, Hamburg, Germany].

The collected data were analysed using the SPSS version 21.0. The level of significance was set as p≤0.05. Variables were analysed using the Chi-square and Fisher's exact tests. Ethical approval from Sudan Medical Specialisation Board research ethics committee was obtained before starting this research and informed consent was obtained from all participants.

## Results

There were no significant differences between the two groups in terms of demographic data (age, sex), fracture type (classification), mode of trauma, comorbidities and mean follow-up duration (Table I). Allocation of patients to any of the two groups depended on the protocol used in the hospital where the treatment was carried out. The duration of surgical operation with hemiarthroplasty was less compared to DHS: 6.7% in the hemiarthroplasty group, compared to 10.5% in the DHS group which needed more than two hours of surgery, though the difference was statistically insignificant (p=0.749).

**Table I:** Main demographic and clinical data

	Hemiarthroplasty	DHS
Mean age (years)+SD	76.15+7.2	79.3+11
Male: Female	23:37	20:18
Fracture type		
Kyle 3	41.7%	26.3%
Kyle 4	58.3%	73.7%
Mode of trauma		
Domestic fall	96.7%	94.7%
Mean follow up+SD	13.66+5.9	11.8+2.7
Co morbidities		
DM	17	5
Asthma	0	1
None	41	32

Regarding intraoperative complications, the need for blood transfusion was less in the DHS group (47.4%) than in the hemiarthroplasty group (61.6%); however, this difference was statistically insignificant (p=0.239). Considering postoperative outcome parameters, patients who underwent hemiarthroplasty had a shorter postoperative hospital stay compared to patients who underwent DHS. In the hemiarthroplasty group, 93.3% of patients, compared to 73.7% in the DHS group, needed hospital stay for less than a week, and the difference between both groups was significant (p=0.010).

All patients in the hemiarthroplasty group were mobilised in bed on the same day of the procedure and 93.3% were able to start partial weight bearing on the first postoperative day. This contrasted with observations in the DHS group where no patient was able to start partial weight bearing on the first postoperative day, and 73.7% were able to start partial weight bearing after 15 days (p<0.001); the rest of this group started partial weight bearing even later. Most patients who underwent hemiarthroplasty (85.2%) started full weight bearing at the end of the first week postoperatively, while patients who underwent DHS started full weight bearing at 6 to 12 weeks postoperatively (p<0.001).

The mean Harris Hip score at 12 weeks postoperatively was 77.85±8.9 for the hemiarthroplasty group and 52.97±16.2 for the fixation group (p=0.001).The final follow-up was 13.66±5.9 months in the hemiarthroplasty group and 11±2.7 months in the DHS group. Of the 60 patients who underwent hemiarthroplasty, ten (16.7%) had died and two (3.3%) were lost to follow-up. Three (5%) patients had infections, two with deep infections that necessitated the removal of the implants; one was left as a girdlestone excision arthroplasty and the other revised later after elimination of the infection. One patient had superficial infection. Two patients (3.3%) had deep venous thrombosis and five (8.3%) had bedsores of whom three had the bedsores before surgery. Only one (1.7%) patient had dislocation of the hip which was reduced surgically (Table II).

**Table II:** Clinical and mechanical complications in the two groups

Complication	Hemiarthroplasty	DHS
Infection	5%	18.4%
Deep	3.3%	5.2%
Superficial	1.7%	13.2%
Deep venous thrombosis	3.3%	5.2%
Bedsore	8.3%	7.9%
Cutout	0	10.5%
Delayed union	0	2.6%
Dislocation	1.7%	0
Non-union	0	2.6%
Malunion		
Varus malunion	0	7.9%
Medialization	0	5.2%
Mortality	16.7%	15.8%

Six of the 38 patients in the DHS group had died (15.8%); five patients were lost to follow-up. Seven patients (18.4%) had complication with infection, of whom five cases were superficial, managed with debridement and antibiotics, and two cases were deep necessitating removal of implants and revision in one case, with external fixation and removal with debridement in the other. There were five patients with general complications (13.2%), two patients had deep venous thrombosis (5.2%) and other three had bedsores (7.9%). Four patients (10.5%) had cut-out and penetration into acetabulum which was revised later with hemiarthroplasty, one patient had non-union revised with hemiarthroplasty, one with delayed union, three cases with varus malunion and two cases ended with medialization (Table II).

The mean Harris Hip score at time of final follow-up was 74.11±13.8 for DHS group and 91.14±5.7 for the hemiarthroplasty group (p<0.001). The reoperation rate was significantly less (p=0.006) in the hemiarthroplasty group: 23.7% of patients in the DHS group, compared to 5% in the hemiarthroplasty group, who needed reoperation.

## Discussion

While many authors have recommended the use of internal fixation in the treatment of unstable intertrochanteric fractures in elderly patients^1-3^, others have recommended prosthetic replacement for treatment of unstable intertrochanteric fractures with improved outcome^7-15^.

The present study showed better results with hemiarthroplasty than internal fixation with DHS for treatment of unstable hip fracture in elderly patients in terms of clinical and functional outcome. The duration of surgery was less in hemiarthroplasty. Huang and Yee reported similar result in their retrospective study comparing DHS (n=72), PFNA (n=43) and hemiarthroplasty (n=16)^16^. Partial and full weight bearing are significantly earlier in the hemiarthroplasty group, as also reported by Kayali *et al* comparing cone hemiarthroplasty (n=42) with DHS (n=24) for unstable hip fractures with a significant earlier full weight bearing in the hemiarthroplasty group^17^. Parker and Handoll in their review of literature in Cochrane database, comparing arthroplasty and internal fixation for unstable extracapsular hip fractures in adults, reported earlier weight bearing in the arthroplasty group^18^. Huang and Yee in their retrospective study reported earlier weight bearing in the hemiarthroplasty group^16^. There was no difference in the mortality rate at one year in hemiarthroplasty and DHS group (17% and 16% respectively) with similar results to Parker and Handoll^18^ and Kayali *et al*^17^. Deep venous thrombosis in both groups were similar (3% and 5% respectively) as reported by Parker and Handoll^18^.

**Fig. 1:**
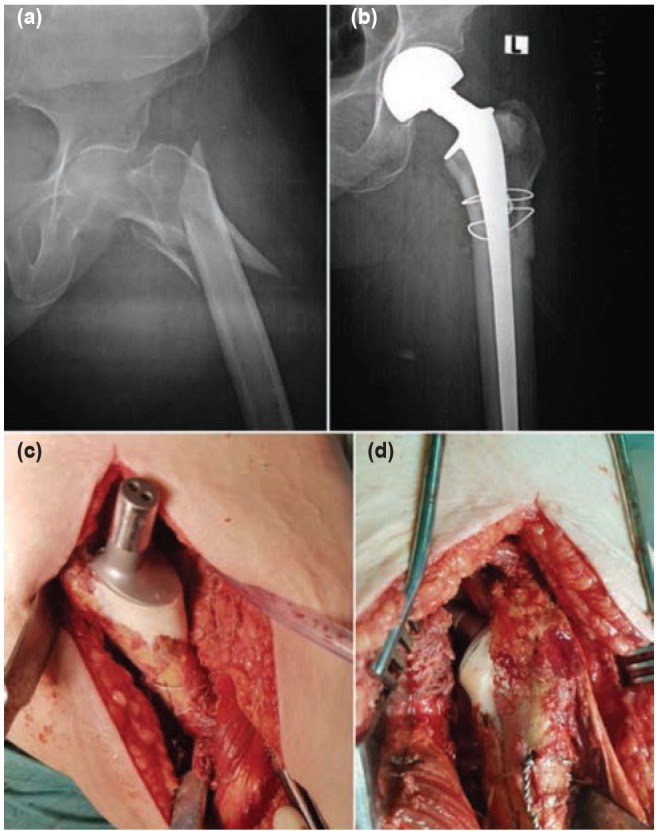
Unstable reverse oblique fracture with (a) Pre- and (b) Post-operative radiographs and intraoperative images showing the reconstruction of the greater trochanter and the cemented stem (c) Before and (d) After reduction.

**Fig. 2:**
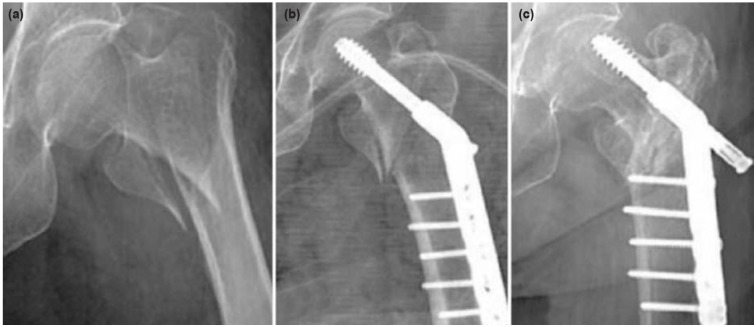
(a) Anteroposterior pre-operative, (b) Post-operative and (c) Final follow-up radiographs showing an unstable intertrochanteric fracture fixed with DHS.

The blood loss was more in hemiarthroplasty group without statistical significance. The reoperation rate was higher in DHS (24% vs 5%). In the DHS group, cut out was around 11%, similar to that reported by Kayali *et al*^17^. The mean final Harris Hip score was significantly higher in hemiarthroplasty group both at three months and the final follow-up (78 vs 53) and (91 vs 74) respectively.

Our limitations were relatively shorter duration of follow-up, lack of randomization and the procedures done in different hospitals by different teams.

## Conclusion

In certain circumstances, the arthroplasty provided more satisfactory outcome than DHS. The main advantage is earlier mobilization which decreases the overall rate of immobility-related complications. The one year mortality rate and DVT were the same in the two groups.

## Conflict of Interest

None of the authors have affiliations with or involvement in any organization or entity with any financial interest or non-financial interest in the subject matter or materials discussed in this study.

## References

[1] Haynes RC, Poll RG, Miles AW, Weston RB (1997). Failure of femoral head fixation: a cadaveric analysis of lag screw cut-out with the gamma locking nail and AO dynamic hip screw. Injury..

[2] Wolfgang GL, Bryant MH, O’Neill JP (1982). Treatment of intertrochanteric fracture of the femur using sliding screw plate fixation. Clin Orthop Relat Res..

[3] Simpson AH, Varty K, Dodd CA (1989). Sliding hip screws: modes of failure. Injury..

[4] Reindl R, Harvey EJ, Berry GK, Rahme E, Canadian Orthopaedic (2015). Trauma Society (COTS). Intramedullary versus extramedullary fixation for unstable intertrochanteric fractures: a prospective randomized controlled trial. J Bone Joint Surg Am..

[5] Tronzo RG (1974). The use of an endoprosthesis for severely comminuted trochanteric fractures. Orthop Clin North Am..

[6] Kyle RF, Cabanela ME, Russell TA, Swiontkowski MF, Winquist RA, Zuckerman JD, Schmidt AH, Koval KJ (1994). Fractures of the proximal part of the femur. J Bone Joint Surg Am..

[7] Harwin SF, Stern RE, Kulick RG (1990). Primary Bateman-Leinbach, bipolar prosthetic replacement of the hip in the treatment of unstable intertrochanteric fracture in the erderly. Orthopedics..

[8] Broos PL, Rommens PM, Deleyn PR, Geens VR, Stappaerts KH (1991). Pertrochanteric fractures in the elderly: Are there indications for primary prosthetic replacement?. J Orthop Trauma..

[9] Chan KC, Gill GS (2000). Cemented hemiarthroplasties for elderly patients with intertrochanteric fractures. Clin Orthop Relat Res..

[10] Stern MB, Angerman A (1987). Comminuted intertrochanteric fractures treated with Lienbach prosthesis. *Clin Orthop Relat Res.*.

[11] Green S, Moore T, Proano F (1987). Bipolar prosthetic replacement for the management of unstable intertrochanteric fractures in elderly. Clin Orthop Relat Res..

[12] Haentjens P, Casteleyen PP, De Boeck H, Handelberg F, Opdecam P (1989). Treatment of unstable intertrochanteric and subtrochanteric fracture in elderly patients. Primary bipolar arthroplasty compared with internal fixation. J Bone Joint Surg Am..

[13] Harvin SF, Stern RE, Kulick RG (1990). Primary Bateman-Lienbach Bipolar prosthetic replacement of the hip in the treatment of unstable intertrochanteric fractures in the elderly. Orthopedics..

[14] Rodop O, Kiral A, Kaplan H, Akmaz I (2002). Primary bipolar hemiprosthesis for unstable intertrochanteric fractures. Int Orthop..

[15] Abdelgadir AH, Awadelsied MH, Elbushra EM, Gashi YN (2016). Outcome of cemented bipolar as primary management of comminuted unstable intertrochanteric fracture femur in elderly Sudanese patients. Univers J Public Health..

[16] Huang CG, Yee JJ (2012). [Comparison of three methods for the treatment of aged femoral intertrochanteric fracture]. Zhongguo Gu Shang..

[17] Kayali C, Agus H, Ozluk S, Sanli C (2006). Treatment for unstable intertrochanteric fracture in elderly patients: internal fixation versus cone hemiarthroplasty. J Orthop Surg (Hong Kong)..

[18] Parker MJ, Handoll HH (2006). Replacement arthroplasty versus internal fixation for extracapsular hip fractures in adults. Cochrane Database Syst Rev..

